# High Q Resonant Sb_2_S_3_-Lithium Niobate Metasurface for Active Nanophotonics

**DOI:** 10.3390/nano11092373

**Published:** 2021-09-13

**Authors:** Qi Meng, Xingqiao Chen, Wei Xu, Zhihong Zhu, Xiaodong Yuan, Jianfa Zhang

**Affiliations:** 1 College of Advanced Interdisciplinary Studies, National University of Defense Technology, Changsha 410073, China; Monky19@163.com (Q.M.); chenxingqiao14@nudt.edu.cn (X.C.); weixu08a@163.com (W.X.); zzhwcx@163.com (Z.Z.); x.d.yuan@163.com (X.Y.); 2 Hunan Provincial Key Laboratory of Novel Nano-Optoelectronic Information Materials and Devices, National University of Defense Technology, Changsha 410073, China

**Keywords:** phase change materials, lithium niobate, metasurface

## Abstract

Phase change materials (PCMs) are attracting more and more attentions as enabling materials for tunable nanophotonics. They can be processed into functional photonic devices through customized laser writing, providing great flexibility for fabrication and reconfiguration. Lithium Niobate (LN) has excellent nonlinear and electro-optical properties, but is difficult to process, which limits its application in nanophotonic devices. In this paper, we combine the emerging low-loss phase change material ${\mathrm{Sb}}_{2}S_{3}$ with LN and propose a new type of high Q resonant metasurface. Simulation results show that the ${\mathrm{Sb}}_{2}S_{3}$-LN metasurface has extremely narrow linewidth of 0.096 nm and high quality (Q) factor of 15,964. With LN as the waveguide layer, strong nonlinear properties are observed in the hybrid metasurface, which can be employed for optical switches and isolators. By adding a pair of Au electrodes on both sides of the LN, we can realize dynamic electro-optical control of the resonant metasurface. The ultra-low loss of ${\mathrm{Sb}}_{2}S_{3}$, and its combination with LN, makes it possible to realize a new family of high Q resonant metasurfaces for actively tunable nanophotonic devices with widespread applications including optical switching, light modulation, dynamic beam steering, optical phased array and so on.

## 1. Introduction

Attracted by the rewritable and non-volatile characteristics of phase change materials (PCMs), extensive works have been done to explore them for tunable optics [1,2,3]. ${\mathrm{Ge}}_{2}{\mathrm{Sb}}_{2}{\mathrm{Te}}_{5}$ (GST) and ${\mathrm{VO}}_{2}$ are probably the commonest PCMs in nanophotonics [4,5,6]. They are widely used in applications such as dynamic thermal emission [7,8,9,10], light modulation [11,12,13,14,15,16,17], beam steering [18,19], polarization conversion [20], colour display [21,22,23] and various tunable metasurfaces and metadevices [24,25,26,27,28,29,30,31,32,33,34,35]. Customized laser has already been used to write, erase, and rewrite GST films into two-dimensional binary or gray-scale functional patterns, which would induce local refractive index changes and construct nanophotonic devices [36]. On the other hand, microheaters using of tungsten (W) or ITO can also be applied to write and erase the patterns in GST, making it possible for programmable nanophotonics [37,38,39]. However, most phase change materials including GST and ${\mathrm{VO}}_{2}$ have unavoidable absorption losses at the near infrared, which limits their further application in photonic devices. Recent research has proposed a new type of PCMs- ${\mathrm{Sb}}_{2}S_{3}$ and ${\mathrm{Sb}}_{2}{\mathrm{Se}}_{3}$ [40], They have low absorption losses of at the near infrared, which can greatly enhance the application of PCMs in photonic devices.

Lithium niobate (${\mathrm{LiNbO}}_{3}$, LN) is one of the most important synthetic crystals and has been dubbed as the “silicon of photonics” for its excellent properties such as relatively high refractive index, wide transparent window, low absorption losses, large nonlinear optical coefficient, outstanding electro-optical response, good temperature stability and others [41,42,43]. Particularly, with the progress in thin-film LN on insulator, it has emerged as promising platform for ultracompact photonic devices, such as low-loss waveguides [44,45], high Q resonators [46,47,48], metasurfaces [49,50], optical modulators [51,52,53,54,55,56], and second harmonic generation [57,58,59,60]. However, LN’s high hardness and inactive chemical properties make its processing difficult, which severely limits its application in nano-devices and integrated optics.

In this paper, we combine phase change material ${\mathrm{Sb}}_{2}S_{3}$ with LN for the first time and propose a type of resonant ${\mathrm{Sb}}_{2}S_{3}$-LN metasurface, where a thin ${\mathrm{Sb}}_{2}S_{3}$ layer locates on top of a LN film and works as a subwavelength refractive index grating. Such a refractive index grating is comprised of periodical distributions of amorphous and crystalline ${\mathrm{Sb}}_{2}S_{3}$, which can be “written”, “erased” or “rewritten” with a customized writing beam (laser or ion). Simulation results show that the resonant ${\mathrm{Sb}}_{2}S_{3}$-LN metasurface has extremely narrow linewidth and high Q factors. The optical spectra can be continuous tuning by not only the duty cycle of the grating, but also the crystallization fraction of the switched ${\mathrm{Sb}}_{2}S_{3}$. Combining with the nonlinear and electro-optical properties of LN, the hybrid metasurface provides unprecedented possibility of active nanophotonics such as nonlinear propagation and electro-optical control.

## 2. Results and Discussion

**Figure 1:**
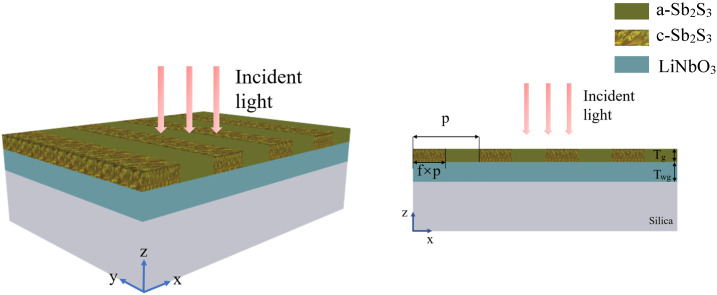
Schematic of a guided mode resonant grating, which consists of a grating layer composed of $c\_{\mathrm{Sb}}_{2}S_{3}$ and $a\_{\mathrm{Sb}}_{2}S_{3}$, a LN waveguide layer and a ${\mathrm{SiO}}_{2}$ substrate of semi-infinite thickness.

With the ultra-low optical absorption and relatively high refractive index, phase change material ${\mathrm{Sb}}_{2}S_{3}$ provides a promising choice for high Q resonant nanophotonics. As is shown in Figure 1, the resonant metasurface is composed of a thin film ${\mathrm{Sb}}_{2}S_{3}$ grating, a LN waveguide layer and a ${\mathrm{SiO}}_{2}$ substrate. After being deposited on the LN waveguide layer, a customized laser pulses or ion beam can be used to write, erase, and rewrite a functional pattern into the phase-change films [36,61]. The ${\mathrm{Sb}}_{2}S_{3}$ can be transformed from amorphous state to crystalline state and vice versa at specific temperature [40]. After part of $a\_{\mathrm{Sb}}_{2}S_{3}$ is converted to $c\_{\mathrm{Sb}}_{2}S_{3}$, the low refractive index $a\_{\mathrm{Sb}}_{2}S_{3}$ and high refractive index $c\_{\mathrm{Sb}}_{2}S_{3}$ form a periodical ${\mathrm{Sb}}_{2}S_{3}$ subwavelength grating, which can effectively scatter free-space incident light and achieve guided mode resonances [62].The flexibility of design enables us to realize various high-Q resonant nanophotonic devices with ${\mathrm{Sb}}_{2}S_{3}$.

The numerical simulations are implemented in a fully three-dimensional finite element technology (in COMSOL Multiphysics). Since our structure is assumed to be “infinitely extending” in the y direction, a two-dimensional model is used for simulations (x-z plane). We use Floquet periodic boundary conditions in the x-direction, and port boundary conditions at the top and bottom of the model along the z-direction. In the simulation, silica (${\mathrm{SiO}}_{2}$) can be regarded as a lossless medium in the near infrared, with a refractive index of $n_{{\mathrm{SiO}}_{2}}=1.45$. The complex refractive indices of $c\_{\mathrm{Sb}}_{2}S_{3}$$n_{c}$ and $a\_{\mathrm{Sb}}_{2}S_{3}$$n_{a}$ are taken from experimental measurement [40], with a negligible loss at the near infrared (see the Supplementary Information Figure S1). For example, the complex refractive indices of $c\_{\mathrm{Sb}}_{2}S_{3}$ and $a\_{\mathrm{Sb}}_{2}S_{3}$ at the wavelength of 1550 nm are $n_{c}$ = 3.308 + 0i and $n_{c}$ = 2.712 + 0i, respectively. For a z-cut LN, we have refractive index of $n_{o}$ = 2.286 and $n_{e}$ = 2.203 [63].

**Figure 2:**
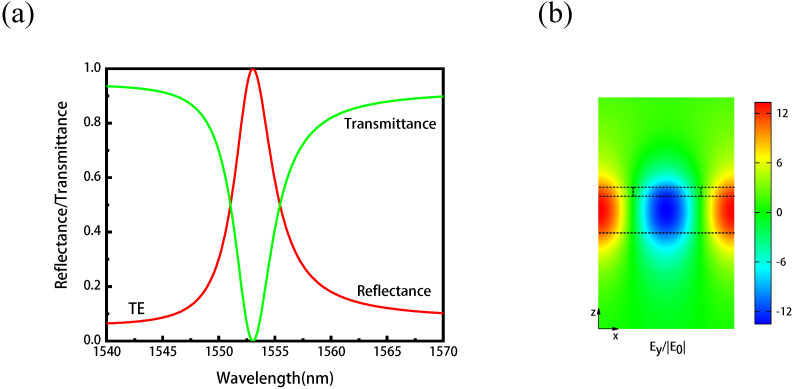
(**a**) The transmission and reflection spectra of the hybrid resonant metasurface. The reflectivity reaches almost 100% around 1553.00 nm. (**b**) The resonant electric field distribution corresponding to the wavelength of 1553.00 nm.

The thickness of the ${\mathrm{Sb}}_{2}S_{3}$ and LN are $T_{g}$ = 40 nm and $T_{\mathrm{wg}}$ = 260 nm, respectively. We set the grating period P = 800 nm and duty cycle *f* = 0.5, and Figure 2a gives the transmission and reflection spectra of the resonant ${\mathrm{Sb}}_{2}S_{3}$-LN metasurface. For a transverse electric (TE) mode with its electric field polarized in the y-direction impinges on the metasurface at normal incidence, a sharp guided mode resonance is excited at the telecom wavelength and the reflectivity reaches nearly 100% at the resonance wavelength of 1553.00 nm. As shown in the Figure 2b, the electric field is mainly distributed in the LN waveguide layer, and the electric field component $E_{y}$ has been enhanced by more than 12 times at the resonance wavelength.

Besides, one can also deposit a ${\mathrm{Sb}}_{2}S_{3}$ layer on a waveguide layer such as silicon (Si), silicon nitride (SiN) or others to achieve high Q resonances. We studied the optical spectra of a guided mode resonant grating structure with ${\mathrm{Sb}}_{2}S_{3}$ on Si. Similar resonances can be observed and the electric field component $E_{y}$ has been enhanced by more than 15 times and the Q factor reaches 744 (see the Supplementary Information Figure S2). The flexibility of design enables us to realize various high-Q resonant nanophotonic devices with ${\mathrm{Sb}}_{2}S_{3}$.

**Figure 3:**
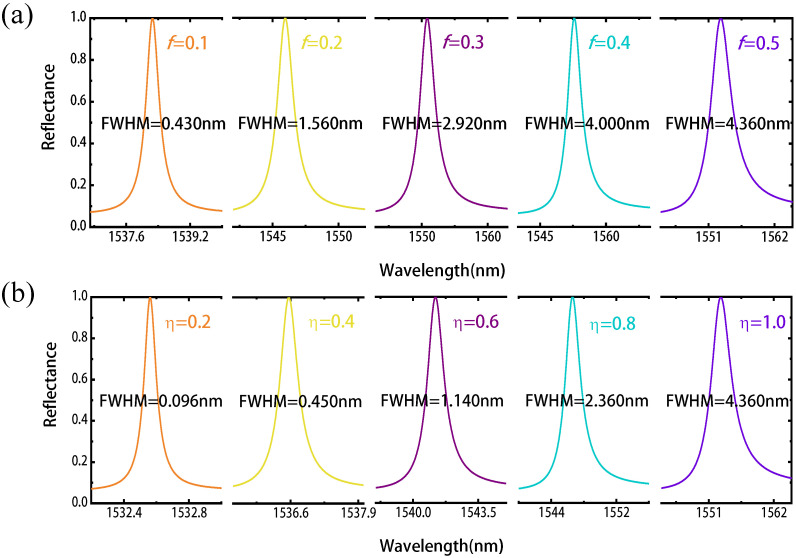
The reflection spectra of the ${\mathrm{Sb}}_{2}S_{3}$-LN metasurface with different duty cycle *f* (with the crystallization fraction η fixed on 1) and different crystallization fraction η (with the duty cycle *f* fixed on 0.5) of the ${\mathrm{Sb}}_{2}S_{3}$ subwavelength grating. (**a**) As the duty cycle *f* decreases from 0.5 to 0.1, the peak of the reflection spectrum shifts to the shorter wavelength, and the FWHM of the structure decreases. (**b**) As the crystallization fraction η decreases from 1 to 0.2, the peak of the reflection spectrum shifts to the shorter wavelength, and the FWHM of the structure decreases.

Benefiting from the ability of continuous tuning of phase change materials, we can achieve flexible control of resonant wavelength by changing the geometric parameters of the metasurface. We first change the duty cycle *f* of the grating and obtain the reflection spectra corresponding to different duty cycles. As shown in Figure 3a, when the duty cycle reduces from f=0.5 to f=0.1, the resonance wavelength shifts from 1553.00 nm to 1538.26 nm, and nearly 100% reflection is achieved. At the same time, the spectral linewidth (full width at half maximum, FWHM) decreases from 4.36 nm to 0.430 nm. Herein, we give the basic relationship between the resonance mode Q factor and FWHM:

(1) $$Q=\frac{\lambda_{r}}{FWHM}$$

where $\lambda_{r}$ denotes the resonant wavelength. Thus, the corresponding Q factor increases from 356 to 3577, with the maximal electric field enhancement increases from 12 to 41 (see the Supplementary Information Figure S3a).

Then, we fix the duty cycle *f* = 0.5, and study the reflection spectra corresponding to different crystalline fractions η of ${\mathrm{Sb}}_{2}S_{3}$. Here, the relationship between the effective dielectric constant of the ${\mathrm{Sb}}_{2}S_{3}$ and the crystalline fraction η is given by the Lorenz-Lorentz relationship [31]:

(2) $$\frac{\varepsilon_{eff}\left(\lambda \right)-1}{\varepsilon_{eff}\left(\lambda \right)+2}=\eta \times \frac{\varepsilon_{c\_Sb_{2}S_{3}}\left(\lambda \right)-1}{\varepsilon_{c\_Sb_{2}S_{3}}\left(\lambda \right)+2}+\left(1-\eta \right)\times \frac{\varepsilon_{a\_Sb_{2}S_{3}}\left(\lambda \right)-1}{\varepsilon_{a\_Sb_{2}S_{3}}\left(\lambda \right)+2}$$

where $\varepsilon_{c\_Sb_{2}S_{3}}\left(\lambda \right)$ and $\varepsilon_{a\_Sb_{2}S_{3}}\left(\lambda \right)$ are the wavelength-dependent permittivity of crystalline and amorphous ${\mathrm{Sb}}_{2}S_{3}$, respectively. And $\varepsilon_{eff}\left(\lambda \right)$ is the effective dielectric constant of the hybridization ${\mathrm{Sb}}_{2}S_{3}$.

As shown in Figure 3b, when the crystalline fraction reduces from η = 1 to η = 0.2, the resonance wavelength shifts from 1553.00 nm to 1532.56 nm, maintaining nearly 100% peak reflection. At the same time, the spectral linewidth decreases from 4.36 nm to 0.096 nm, and the Q factor increases from 356 to 15,964, with the maximal electric field enhancement increase from 12 to 90 (see the Supplementary Information Figure S3b). The increase of Q factors is attributed to the reduction of scattering, i.e., coupling with the incident light with the decrease of refractive index contrast in the ${\mathrm{Sb}}_{2}S_{3}$ grating. These high Q resonances are possible as both ${\mathrm{Sb}}_{2}S_{3}$ and LN show very low losses at the telecom wavelength, which is hardly achievable for traditional phase change materials such as GST or ${\mathrm{VO}}_{2}$.

### 2.1. Nonlinear Optics with the $Sb_{2}S_{3}$-LN Resonant Metasurface

Since the field enhancement of the structure is very considerable, we now consider the nonlinear properties of the resonant ${\mathrm{Sb}}_{2}S_{3}$-LN metasurface to explore the strong light-matter interaction. The nonlinear Kerr effect describes the change in dielectric constant of the medium caused by the interaction of the external field and the third-order nonlinear susceptibility, and can be expressed as [64]:

(3) $$\varepsilon \left(r\right)=\varepsilon_{0}\left(\varepsilon_{r}+\chi^{\left(3\right)}{\left|E_{r}\right|}^{2}\right)$$

where $\chi^{\left(3\right)}$ denotes the third-order nonlinear susceptibilities and $|E_{r}|$ denotes the strength of local electric field. For LN, the third-order susceptibility $\chi^{\left(3\right)}$ can be obtained from:

(4) $$\chi^{\left(3\right)}=\frac{4{n_{0}}^{2}n_{2}}{3Z_{0}}$$

where $n_{2}=1.44\times 10^{-15}$ m${}^{2}$⁄W is the third-order nonlinear coefficient of LN [65], $Z_{0}=377$Ω is the vacuum impedance, thus the third-order nonlinear susceptibility of LN is $\chi^{\left(3\right)}=2.66\times 10^{-17}$ m${}^{2}$/V${}^{2}$. And since the third-order nonlinear susceptibility of ${\mathrm{Sb}}_{2}S_{3}$ ($∼10^{-19}$ m${}^{2}$/V${}^{2}$) is much smaller than that of LN, we do not take it into account in our simulation [66].

**Figure 4:**
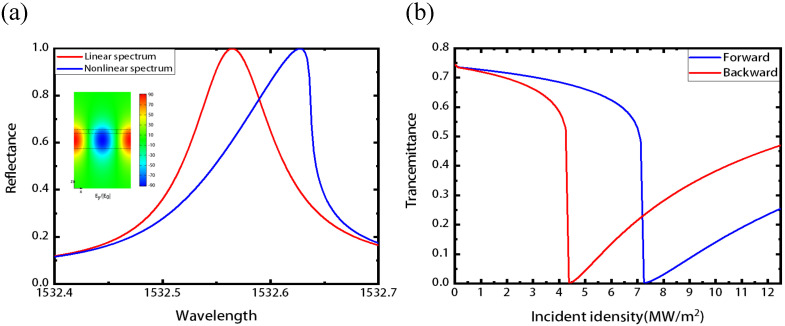
The non-linear and non-reciprocal propagation of the resonant ${\mathrm{Sb}}_{2}S_{3}$-LN metasurface caused by Kerr effect. (**a**) The reflection spectrum of the ${\mathrm{Sb}}_{2}S_{3}$-LN metasurface with incident intensity of 5.000 MW/m${}^{2}$. Red and blue curves represent linear and nonlinear spectra, respectively. Inset is electric field enhancement of the ${\mathrm{Sb}}_{2}S_{3}$-LN metasurface with grating parameters of f = 0.5 and η = 0.2. (**b**) The non-reciprocal curve with the wavelength fixed at 1532.654 nm. The backward transmission is 0 with incident intensity of 4.375 MW/m${}^{2}$, while the forward is 0.67 with the same incident intensity.

Due to the high nonlinear coefficient of LN and the strong electric field enhancement inside the LN waveguide layer, the nonlinear effect can be easily observed in the resonant metasurface. In order to obtain obvious nonlinear effect results under lower modulation power, we use the ultra-high Q factor structure parameters, with *f* = 0.5, η = 0.2, and other parameters are consistent with those in Figure 1. The enhancement of fields is shown in inset of Figure 4a. The reflection spectrum of the ${\mathrm{Sb}}_{2}S_{3}$-LN metasurface is given in the Figure 4a with an incident intensity of 5.000 MW/m${}^{2}$, and the nonlinear spectrum is red-shifted from the resonance wavelength of 1532.564 nm to 1532.628 nm due to the decrease of the overall equivalent permittivity of the LN (the third-order susceptibility of LN is positive number). As an application of the nonlinear optical response, we can design a nonlinear optical isolator considering the asymmetry of the structure in the light propagation direction. Figure 4b gives the nonlinear non-reciprocal curves for forward and backward incident light at 1532.654 nm. With the intensity of incident light increases, the forward and backward incident light have different transmission spectra: the transmissivity of backward incident light reaches to 0 at 4.375 MW/m${}^{2}$, while that of forward is 0.67. The non-reciprocal can be adjusted through varying the parameters of the ${\mathrm{Sb}}_{2}S_{3}$ grating and can be obtained at the desired wavelength [64]. Besides, the proposed resonant metasurface may also be explored for other nonlinear optical applications such as second harmonic generation [67,68].

Previous experiments [40] have shown that $c\_{\mathrm{Sb}}_{2}S_{3}$ and $a\_{\mathrm{Sb}}_{2}S_{3}$ can exist stably under the light intensity of thousands of MW/m${}^{2}$, and in our work, the maximum intensity of the near-infrared light we use is under 12 MW/m${}^{2}$. Meanwhile, only a small part of the near-infrared light used in the study of nonlinearity and non-reciprocity will be absorbed by ${\mathrm{Sb}}_{2}S_{3}$. Thus, the ${\mathrm{Sb}}_{2}S_{3}$ in our structure can tolerate the incident intensity mentioned above and achieve the expected nonlinear and non-reciprocal effects.

### 2.2. Electro-Optical Tunability of the $Sb_{2}S_{3}$-LN Resonant Metasurface

**Figure 5:**
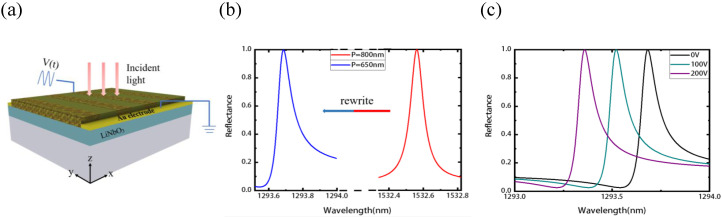
The electro-optical tunability of the resonant ${\mathrm{Sb}}_{2}S_{3}$-LN metasurface. (**a**) The schematic of the resonant metasurface with a pair of Au electrodes. (**b**) The phase change reconfiguration of the resonant metasurface. As the grating period rewritten from P = 800 nm to P = 650 nm, the resonant wavelength shifts from 1532.564 nm to 1293.682 nm. (**c**) The reflection spectra vary with different gate voltage. As the voltage adds from 0 V to 200 V, the resonant wavelength shifts from 1293.682 nm to 1293.358 nm.

Now, we turn to investigate the electro-optical properties of the resonant ${\mathrm{Sb}}_{2}S_{3}$-LN metasurface. As shown in the Figure 5a, we now add a pair of Au electrodes at the side of the ${\mathrm{Sb}}_{2}S_{3}$ grating (electrical isolation between the electrodes and phase change material can be realized with an insulator layer when necessary). The length along the y direction of the ${\mathrm{Sb}}_{2}S_{3}$ grating is set as L = 10 μm, which is much larger than that of the resonant wavelength (it is regard as infinite in the y direction in simulations for simplicity). To demonstrate the super reconfiguration ability of the proposed structure, we first tune the resonant wavelength as an example of rewriting the ${\mathrm{Sb}}_{2}S_{3}$ grating by varying the period from P = 800 nm to P = 650 nm, with *f* = 0.5, η = 0.2 (the high Q parameters), and other parameters are same with that of in Figure 1. As we rewrite the period to be P = 650 nm, the resonance wavelength moves from 1532.564 nm to 1293.682 nm, as shown in Figure 5b. We next add gate voltage from the electrode in x-direction of the LN for dynamic electro-optical tuning, and thus the change of $n_{o}$ along with the voltage can be described that [69]:

(5) $$n_{o}^{{}^{\prime }}=n_{o}-\frac{1}{2}r_{22}{n_{o}}^{3}E_{0y}$$

where $r_{22}=6.8$ pm/V is the electro-optic coefficient of LN and $E_{0y}$ is the electric field applied to the LN layer (along with the Y axis of LN).

As we add different gate voltages, $n_{o}$ will vary along with the gate voltage (see the Supplementary Information Figure S5), and thus we can tune the reflection spectra of the ${\mathrm{Sb}}_{2}S_{3}$-LN metasurface with the electric signal. As shown in the Figure 5c, with the gate voltage increases from 0 V to 200 V, the resonant wavelength shifts from 1293.682 nm to 1293.358 nm, while maintaining 100% reflection. Such a shift is 2.79 times larger than the FWHM of the resonance and thus enough to induce vivid change of the optical spectra. It should be mentioned that for a larger electro-optic coefficient of $r_{33}=33$ pm/V, the gate voltage will be much lower for the same effects.

## 3. Conclusions

In summary, we have demonstrated a type of resonant ${\mathrm{Sb}}_{2}S_{3}$-LN metasurface and shown its promising applications in active nanophotonics. The proposed structure includes a subwavelength grating layer composed of low-loss phase change material ${\mathrm{Sb}}_{2}S_{3}$ and a waveguide layer composed of LN. We can deposit the phase change material ${\mathrm{Sb}}_{2}S_{3}$ on LN, and then use customized laser pulses to realize the required structure [36] (e.g., the grating structure), which overcomes the difficulty of processing of LN in nanoscale. Numerical simulations indicate that the resonant metasurface shows considerable nonlinear and electro-optic effects. In addition, the optical spectra can be continuously adjusted with lithography-free method, as the different duty cycle and the crystalline fraction can be achieved by the laser or ion beam. Other methods, such as electrothermal switching [15,35], could also be applied to induce the crystallization and amorphization of phase change materials.The optical switching of chalcogenide phase change materials can be realized at the nanosecond scale and previous work has demonstrated tuning of phase change optical devices at the speed of tens of MHz [17]. For the practical application of our proposed device, the patterns in the phase change material will be fixed unless one wants to rewrite the structure to change its working wavelength. And the dynamic electro-optical modulation or nonlinear effects can be realized by the LN, whose intrinsic response can be as fast as ∼fs and previous work has demonstrated LN modulators up to 100 GHz [50]. Represented by ${\mathrm{Sb}}_{2}S_{3}$, other ultra-low loss of PCMs in the near infrared, such as ${\mathrm{Sb}}_{2}{\mathrm{Se}}_{3}$ [40] or ${\mathrm{Ge}}_{2}{\mathrm{Sb}}_{2}{\mathrm{Se}}_{4}{\mathrm{Te}}_{1}$ (GSST) [70], can also be applied to combining with LN, makes it possible to realize a new family of high Q resonant metasurfaces for active nanophotonic devices with widespread applications including optical switches, light modulation, dynamic beam steering, optical phased array, optical artificial network and so on.

## Data Availability

The data presented in this study are available on request from the corresponding author.

## Supplementary Materials

The following are available online at https://www.mdpi.com/article/10.3390/nano11092373/s1, Figure S1: The complex refractive index of ${\mathrm{Sb}}_{2}S_{3}$, taken from experimental measurement, Figure S2: Resonant metasurface with a Si waveguide layer. (a) Schematic of the resonant metasurface with a Si waveguide layer, which consists of a grating layer composed of $c\_{\mathrm{Sb}}_{2}S_{3}$ and $a\_{\mathrm{Sb}}_{2}S_{3}$, a Si waveguide layer and a ${\mathrm{SiO}}_{2}$ substrate of semi-infinite thickness. (b) The transmittance and reflectance spectra. The reflectivity reaches almost 100% around 1548.96 nm. (c) The electric field distribution corresponding to the resonance wavelength of 1548.96 nm, Figure S3: Corresponding Q factor and electric filed enhancement with different duty cycle and crystallization fraction. (a) The Q factor and electric filed enhancement with different duty cycle, with the crystallization fraction η fixed on 1. (b) The Q factor and electric filed enhancement with different crystallization fraction, with the duty cycle *f* fixed on 0.5, Figure S4: The change of $n_{o}^{{}^{\prime }}$ along with different gate voltage.
