# Supplementary material for: High Q Resonant Sb2S3-Lithium Niobate Metasurface for Active Nanophotonics
Source: Nanomaterials (Basel). 2021 Sep 13;11(9):2373. doi: 10.3390/nano11092373 (PMC8468812; doi:10.3390/nano11092373)
Supplement: Supplementary file 1 [file nanomaterials-11-02373-s001.zip › nanomaterials-1368408-supplementary.pdf]

Supporting information

High Q Resonant Sb_2S_3-Lithium Niobate Metasurface for Active Nanophotonics

Qi Meng [1,2], Xingqiao Chen [1,2], Wei Xu [1,2], Zhihong Zhu [1,2], Xiaodong Yuan [1,2] and Jianfa Zhang [1,2*]

1 College of Advanced Interdisciplinary Studies, National University of Defense Technology, Changsha 410073,China; Monky19@163.com (Q.M.); chenxingqiao14@nudt.edu.cn (X.C.); weixu08a@163.com (W.X.); zzhwcx@163.com (Z.Z.); x.d.yuan@163.com (X.Y.)

2 Hunan Provincial Key Laboratory of Novel Nano-Optoelectronic Information Materials and Devices, National University of Defense Technology

* Correspondence: Correspondence: jfzhang85@nudt.edu.cn

S1. The complex refractive of Sb_2S_3

The complex refractive indices of c_Sb_2S_3 n_c and a_Sb_2S_3 n_a are given in Fig.S1. For near infrared wavelength, the loss of Sb_2S_3 is negligible.

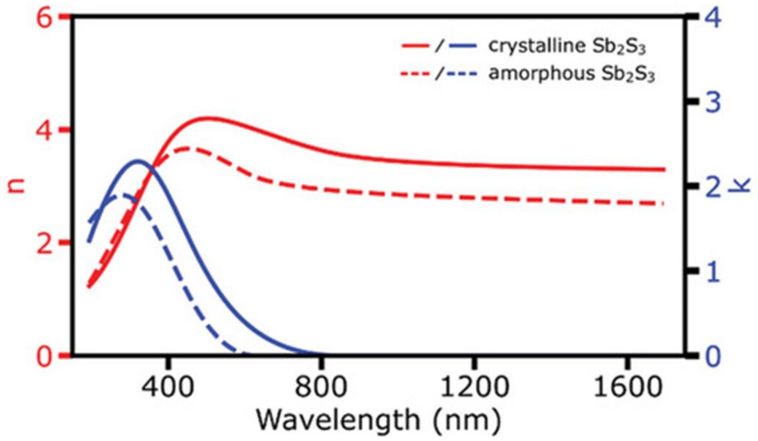

Figure S1. The complex refractive index of Sb_2S_3, taken from experimental measurement[1].

S2. Resonant metasurface with a Si waveguide layer

We studied the optical spectra of a resonant metasurface with a Sb_2S_3 on Si layer. As the shown in Fig.S2a (side view of the structure), the structure consists of a grating composed of c_Sb_2S_3 and a_Sb_2S_3, a Si waveguide layer and a SiO_2 substrate of semi-infinite thickness. In order to obtain the resonance reflection spectrum around 1550nm, we adjusted the structural parameters appropriately, with the thickness of the Sb_2S_3 and Si is T_g=40nm and T_{wg}=205nm, respectively, and the grating period is p=550nm, with the duty cycle is f=0.5. The Si also is regarded as a lossless medium in the near infrared, with a refractive index of n_{Si}=3.42.

Fig.S2b is the transmittance and reflectance spectra of the resonant metasurface. With a waveguide layer, the electric field component E_y has been enhanced by more than 15 times and the Q factor reaches 744.

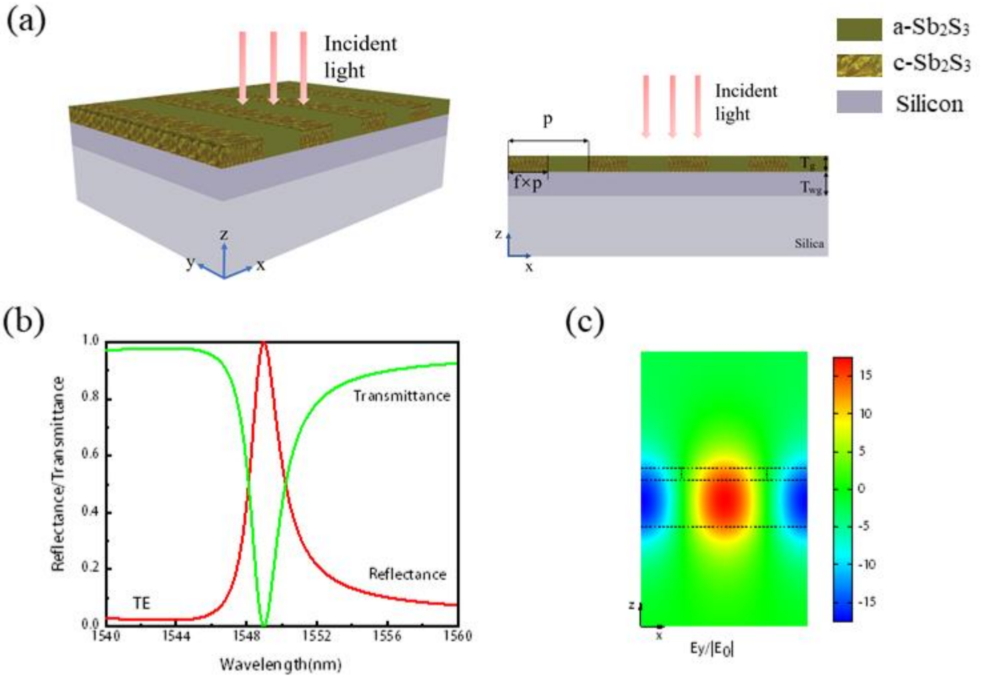

Figure S2. Resonant metasurface with a Si waveguide layer. (a) Schematic of the resonant metasurface with a Si waveguide layer, which consists of a grating layer composed of c_Sb_2S_3 and a_ Sb_2S_3, a Si waveguide layer and a SiO_2 substrate of semi-infinite thickness. (b) The transmittance and reflectance spectra. The reflectivity reaches almost 100% around 1548.96 nm. (c) The electric field distribution corresponding to the resonance wavelength of 1548.96nm.

S3. Corresponding Q factor and electric filed enhancement with different duty cycle and crystallization fraction

As shown in the Fig.S3a, with the duty cycle f decrease from 0.5 to 0.2, the Q factor increased from 356 to 3577, along with increase of maximal electric field enhancement from 12 to 4. Such result keeps path with that of different crystallization fraction, as shown in the Fig.S3b.

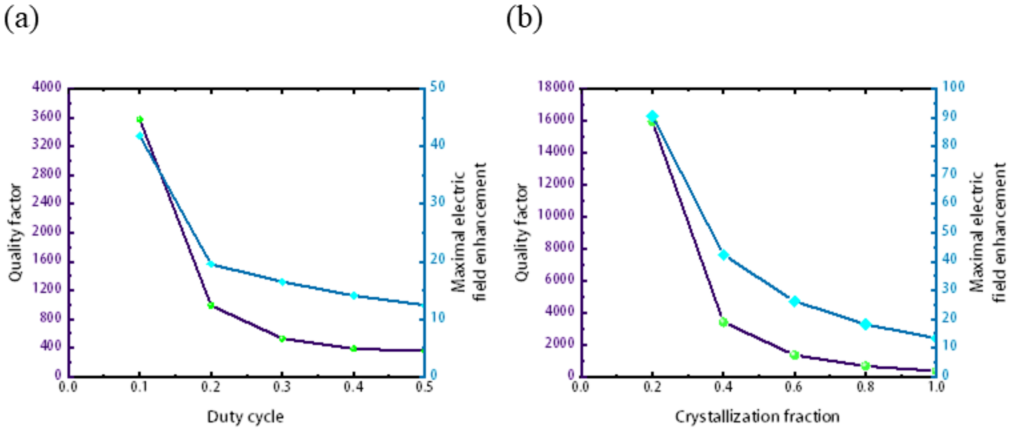

Figure S3. Corresponding Q factor and electric filed enhancement with different duty cycle and crystallization fraction. (a)The Q factor and electric filed enhancement with different duty cycle, with the crystallization fraction η fixed on 1. (b)The Q factor and electric filed enhancement with different crystallization fraction, with the duty cycle f fixed on 0.5.

S4. The change of n_o along with different gate voltage

LN is one of the most important electro-optical crystals. The refractive index of LN can be tuned by an external voltage and here we explore this effect for electro-optical control of the resonant metasurface. As shown in the Fig. **S5,** with the gate voltage adding from 0 to 200V, the n_o' decreases from 2.286 to 2.2852.

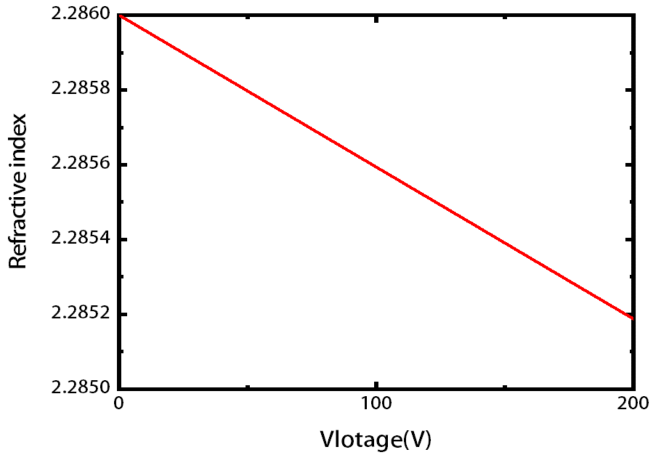

Figure S4. The change of n'_o along with different gate voltage.

References

1. 1. M. Delaney, I. Zeimpekis, D. Lawson, D. W. Hewak and O. L. Muskens, *Advanced Functional Materials*, 2020, **30**, 2002447.
